# Analysis of Concentration Polarisation in Full-Size Spiral Wound Reverse Osmosis Membranes Using Computational Fluid Dynamics

**DOI:** 10.3390/membranes11050353

**Published:** 2021-05-10

**Authors:** Wenshu Wei, Xiang Zou, Xinxiang Ji, Rulin Zhou, Kangkang Zhao, Yuan Wang

**Affiliations:** 1 Beijing Tianma Electro-Hydraulic Control System Company Ltd., Beijing 100013, China; wws@tdmarco.com (W.W.); zhourl@tdmarco.com (R.Z.); zhangkk@tdmarco.com (K.Z.); 2 UNSW Centre for Transformational Environmental Technologies, Yixing 214200, China; xiang.zou@unswctet.com (X.Z.); xinxiang.ji@student.unsw.edu.au (X.J.); 3 School of Civil & Environmental Engineering, UNSW Sydney, Sydney 2052, Australia

**Keywords:** spiral wound module, reverse osmosis, RO, feed spacer, concentration polarisation, computational fluid dynamics, CFD, desalination

## Abstract

A three-dimensional model for the simulation of concentration polarisation in a full-scale spiral wound reverse osmosis (RO) membrane element was developed. The model considered the coupled effect of complex spacer geometry, pressure drop and membrane filtration. The simulated results showed that, at a salt concentration of 10,000 mg/L and feed pressure of 10.91 bar, permeate flux decreased from 27.6 L/(m^2^ h) (LMH) at the module inlet to 24.1 LMH at the module outlet as a result of salt accumulation in the absence of a feed spacer. In contrast, the presence of the spacer increased pressure loss along the membranes, and its presence created vortices and enhanced fluid velocity at the boundary layer and led to a minor decrease in flux to 26.5 LMH at the outlet. This paper underpins the importance of the feed spacer’s role in mitigating concentration polarisation in full-scale spiral wound modules. The model can be used by both the industry and by academia for improved understanding and accurate presentation of mass transfer phenomena of full-scale RO modules by different commercial manufacturers that cannot be achieved by experimental characterization of the mass transfer coefficient or by CFD modelling of simplified 2D flow channels.

## 1. Introduction

Reverse osmosis (RO) has been widely used in desalination, water purification, reclaimed water recovery and industrial wastewater treatment while concentration polarisation (CP) remains a challenge that causes increases in feed pressure, energy consumption, permeate salt concentration and membrane fouling [1,2]. The analysis of concentration polarisation is often based on the use of the Boundary Layer Film Model [3] which relies on experimental or mathematical determination of the mass transfer coefficient of feed solute and the boundary layer thickness, which is difficult to assess for commercial spiral wound modules with spacers. In addition, CP factors calculated from the Boundary Layer Film Model cannot be used to represent the variation in CP and flux change along the axial direction of the membranes. The effect of pressure drops, which affects the feed pressure along different locations in the axial direction and subsequently affects the mass transfer of ions in the boundary layer, cannot be fully captured by the Boundary Layer Film Model. 

Computational fluid dynamics (CFD) is a powerful tool for the investigation of flow behaviour inside water channels and has been widely used to study the effect of spacers on mass transfer and flow behaviour [4,5,6], particle colloid contamination distribution and bacterial film distribution inside the feed channel of RO membranes [7,8]. Schwinge and Wiley et al. [9,10,11] conducted 2D simulations of three typical spacer configurations and discovered that the wall shear stress increased by either decreasing the spacer spacing or increasing the Reynolds number. At the same Reynolds number, turbulent wakes appeared more readily by decreasing the critical Reynolds number (Re_CR_), which were beneficial for reducing contaminant accumulation at the membrane surface and for mitigating the CP phenomenon at a cost of increasing the pressure drop across the membrane module, which further caused an increase in energy consumption. Ahmad et al. [12] performed CFD simulations of circular, triangular and rectangular spacer filaments at the same cross-membrane pressure condition and found that in comparison to rectangular filament, circular and triangular filaments could produce vortices at lower Reynolds numbers. The CP factor of circular filaments was smaller than that of the rectangular filament at the same Reynolds number. Cao et al. [13] used CFD to simulate the effect of the spacing of spacers on fluid flow inside the RO membrane flow channel. The simulation showed that, when the spacer spacing decreased, the distance between the shear stress peak values at the RO membrane surface decreases, i.e., the peaks appeared more frequently, which improved the mass transfer capacity of the membrane. However, this simultaneously increased the pressure drop, thus increasing the energy consumption. On the other hand, when the spacer spacing increased, the pressure drops decreased while the distance between the shear stress peak values at the membrane surface became larger, i.e., the peaks appeared less frequently, thus aggravating the CP phenomenon. Therefore, the study suggested that the mass transfer capacity and energy consumption should both be considered in the selection of optimal spacer spacing. Steady-state laminar and turbulent flow models were used by Ranade and Kumar [14] to simulate the fluid flow with Reynolds numbers between 50 and 1500. The k-ε model was used as the turbulent flow model, and the effect of several optimized spacer designs on fluid flow inside the flow channel was studied in detail; this includes the cylindrical and curvilinear spacer designs. Koutsou et al. [15,16] used a non-steady-state laminar flow model to perform CFD simulations of fluid flow and mass transfer with Reynolds numbers between 70 and 740. The study focused on investigating the effect of different internal angles and the angle of the filaments on fluid flow in the membrane flow channel across several spacer designs. Ruiz-García and Pestana developed [17] a 2D model to simulate the CP of three different Dupont Filmtec RO membrane elements at different salt concentrations, feed flowrates and feed pressure. This model, however, ignored the spatial distribution of salts on the membrane surface of the spiral wound module. 

Three-dimensional CFD models have been developed to investigate the effect of geometrical characteristics of spacers on the performance of RO membranes [18]. Abdelbaky et al. [19] performed CFD analysis on RO membrane modules with feed spacers having fixed or variable diameter under different inlet salinity and Reynolds number conditions. The results indicated that the RO membrane modules with variable-diameter feed spacers can reduce CP. Toh et al. [20] modelled the installed perforations with various locations, diameters and number of perforations on traditional feed spacers and its impact on the hydraulic and mass transport. Advanced characterisation techniques such as Particle Image Velocimetry (PIV) [21] and in situ microscopic observations have also been used to examine the flow behaviour in 3D feed channels incurred by spacers [22].

As the full-size spiral wound RO membrane module has a much more complex geometry and is several orders of magnitude larger than a crossflow flat sheet flow channel in size, most simulations have primarily focused on a single feed spacer or a few feed spacers [23] in order to reduce computational workload. This results in being unable to reveal the hydraulic performances of the full-scale RO membrane module. Moreover, most of the studies neglected permeate flow. Although the impact of permeate on CP can be considered minimal, these models cannot be used to predict the full profile and spatial distribution of flux along the axial direction of the spiral wound membrane modules. 

This paper established a 3D CFD model to simulate a full-scale commercial spiral wound RO (SWRO) membrane module (Dupont Filmtec-BW30-400). Previous work performed by Gu et al. developed a one-dimensional model capturing the spiral characteristics of SWRO membranes by coupling values of geometric parameters such as flow path length, variation of the flow channel height and cross-sectional area [24]. The current work further developed the spiral equation used in the work by Gu et al. by adding the topologies of the feed spacer to establish a complete 3D geometric model of the spiral wound membrane module. The effects of the feed spacer on flow distribution, mass transport and CP were analysed utilizing the comparison of RO modules with a feed spacer and RO modules without a feed spacer. The spatial distribution of flux along the spiral wound membranes in the absence and presence of the spacer was calculated. 

## 2. Model Development and Validation

### 2.1. Governing Equations

CFD simulations of RO processes involve modelling of the mass transfer of the solute (inorganic salts) and solvent (water) at the feed channel and permeate channels, respectively [25,26]. The governing equations for fluid flow are the conservation of mass and conservation of momentum equations for incompressible fluids (Navier–Stokes equations):

(1) $$\nabla \cdot \left(\rho u\right)=S_{v}$$

(2) ∇·(ρuu)=−∇p+∇·(μ∇u)

(3) $$\nabla \cdot \left(\rho um\right)=\nabla \cdot \left(\rho D\nabla m\right)+S_{m}$$

where u denotes the fluid velocity vector, p denotes the pressure, ρ denotes the fluid density, μ denotes the fluid viscosity and m denotes the solute mass fraction. At the feed side of a RO membrane, solvent (water) permeation through RO membranes produces a mass sink term $S_{m}$ (4) and a momentum source term $S_{v}$ (5):

(4) $$S_{m}=-\frac{J\cdot a}{V}$$

(5) $$S_{v}=-\frac{J\cdot a\cdot v}{V}$$

where a is the effective membrane area for solution passage (m^2^); V denotes the corresponding effective volume in the computational domain (m^3^); v denotes the flow velocity perpendicular to the membrane surface (m/s); and J represents the permeate flux of the corresponding solution (m^3^/(m^2^·s)), represented by (6) according to the Kedem–Katchalsky Method:

(6) J=A×(∆P−∆Π)

where A is the permeability coefficient of water through the RO membrane (m/(s·Pa)), ΔP denotes the pressure difference between the two sides of the membrane (Pa) and ΔΠ is the osmotic pressure difference caused by the salt concentration difference between both sides of the membrane. 

The transport of inorganic salts within the entire flow field is expressed through the mass conservation Equation (7):

(7) $$\nabla \cdot \left(uc\right)=\nabla \cdot \left(D\nabla c\right)+S_{s}$$

where c denotes the concentration of the corresponding inorganic salt, u is the velocity of water flow and D is the diffusion coefficient of the corresponding organic salt. $S_{s}$ is the solvent mass source sink term and is calculated as follows:

At the feed channel:

(8) $$S_{s}=0,$$

On the membrane feed surface:

(9) $$S_{s}=-\frac{J_{s}\cdot a}{V}$$

where $J_{s}$ corresponds to the permeate flux of inorganic salt j (kg/(m^2^·s)), which is represented by (10):

(10) $$J_{s}=J\times C_{p}=B\times \left(C_{m}-C_{p}\right)$$

where B is the permeability coefficient of the corresponding inorganic salt through the RO membrane (m/s), and $C_{m}$ and $C_{p}$ represent the concentration of the corresponding inorganic salt on the feed side and on the permeate side of the membrane, respectively (kg/m^3^).

### 2.2. Geometry

#### 2.2.1. Cross-Flow Flat Sheet Module

**Figure 1:**
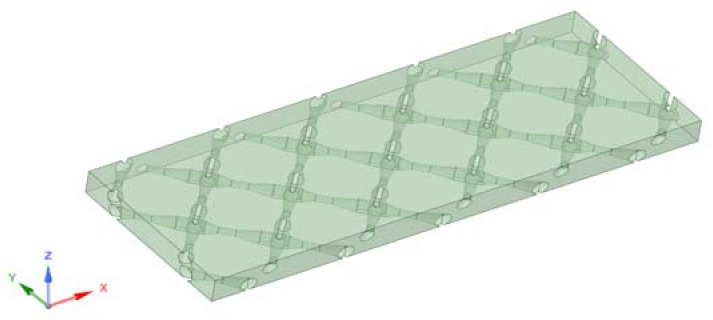
Computational domain of the feed channel.

**Figure 2:**
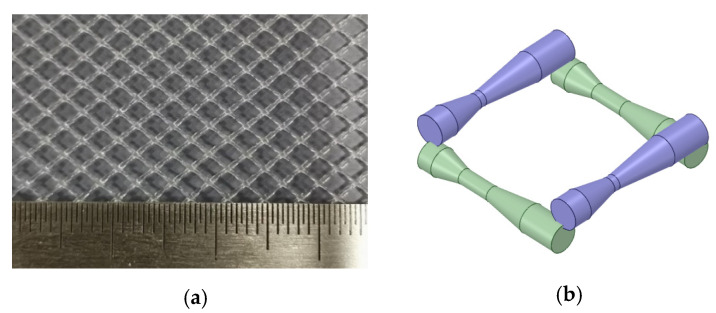
Physical geometries and 3D CAD model of the spacer filament. (**a**) Physical geometries of the spacer filament; (**b**) Three-dimensional CAD model of the spacer filament.

A 3D CFD model was developed to simulate the flow and salt concentration in a 16.86 mm × 6 mm × 0.86 mm (L × W × H) RO cell domain (Figure 1). The feed spacer consisted of fine filaments with varying thicknesses from 0.22 mm to 0.45 mm (Figure 2a,b). Grid inflations at 0.02 mm thickness were applied for the liquid–membrane interfaces, which ensures CP and transport phenomena that occurred were simulated using a higher mesh resolution. A grid size of 0.05 mm was employed universally across the remainder of the domain and resulted in approximately 7,293,005 elements (determined by mesh independence test from 3,000,000 to 15,000,000 elements).

#### 2.2.2. Geometry of a Full-Size RO Membrane Module

**Figure 3:**
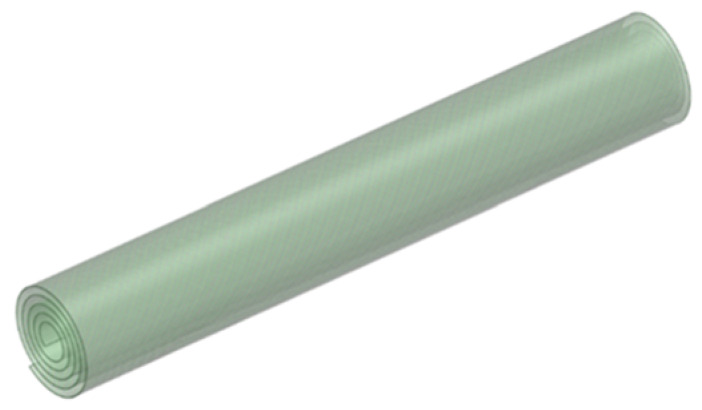
Geometries of the simulated SWRO membrane feed channel.

A full-size spiral wound RO membrane module (Dupont Filmtec-BW30-400) consisting of 10 membrane sheets that were 2.05 m long and 0.9 m wide (with an effective membrane area of 400 ft^2^) and wounded along an Archimedes spiral [27] was modelled in this work. Adjacent membrane sheets shared a common feed channel and ran parallel with one another with some independency; hence, the module’s geometries could be simplified to possess only a single feed channel for the simulation (Figure 3). The geometry of a single feed channel was created using ANSYS SpaceClaim (2019R3) (Ansys, Inc., Canonsburg, PA, USA). Looking down from the axial direction, the Archimedean spiral for the single feed channel rolled up along the length of the membranes had the following equations:

(11) x(θ)=(α+βθ)cos(θ)

(12) y(θ)=(α+βθ)sin(θ)

where x and y are the coordinates of points on the spiral, θ is the angle variable and α and β are constants. The constant α is the distance from the start of the spiral to the vertical axis, i.e., the radius of the tube and the constant β is the ratio between the radial expansion speed of the spiral to its rotational angular speed, which is dependent on the number of wounded membrane sheets, the thickness of the membrane sheets and the thickness of the corresponding feed channel. For the Dupont Filmtec-BW30-400 RO membranes, the corresponding α and β values were 0.02 m and $\frac{0.0062}{\pi }$ m, respectively. The measured thickness of feed spacers, i.e., the feed channel width was 0.86 mm. 

The simulated geometries were meshed using ANSYS Meshing (2019R3) (Ansys, Inc., Canonsburg, PA, USA). In order to accurately simulate the CP phenomenon of inorganic salt contents in the boundary layer, five hexahedral inflation layers that were parallel to the membrane surface were created. The first layer had a height of 0.02 mm and a growth rate of 1.5 for each of the following layers. The rest of the geometries were meshed using tetrahedral elements with an average mesh size of 0.05 mm. Approximately 13 million elements were present after passing the mesh independence test. 

### 2.3. Model Setup

The CP of the RO membrane module in the absence and presence of a feed spacer was simulated using the commercial CFD package ANSYS CFX (2019R3) (Ansys, Inc., Canonsburg, PA, USA) with 10,000 ppm NaCl as feed solution. For the full-size RO module simulation, three different NaCl concentrations were modelled. The physical properties of water were used as the impact of salt concentrations on fluid physical properties, such as viscosity and density, were negligible. The feed inlet was set as the velocity inlet, and the feed outlet was set as the pressure outlet. The surface of the feed spacer was set as a non-slip boundary [28]. The k-ω model was used to model liquid flow. A high-resolution scheme was used to solve for fluid flow control equations. The membrane permeability coefficient A (9.56 × 10^−12^ m/(s·Pa)) obtained from Dupont Filmtec-BW30-400’s published data and salt (NaCl) permeability coefficient *B* (5.58 × 10^−8^ m/s) determined by utilizing experimental measurement at various feed concentrations and pressure [17] were used in this study. All simulated flow velocities and pressures converged to 10^−4^ and inorganic salt concentrations converged to 10^−6^.

### 2.4. Model Validation

**Figure A1:**
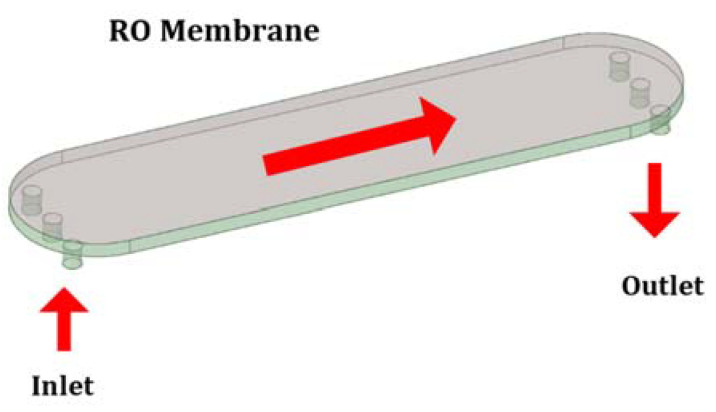
Schematic diagram of the crossflow RO test apparatus for model validation.

A small crossflow RO membrane test apparatus was established for the validation of CFD models. The feed channel had a length of 145.0 mm, a width of 35.0 mm and a height of 3.5 mm with an effective membrane area of 48.12 cm^2^ (Figure A1). Simulation of the test apparatus was conducted using the aforementioned methods. By changing the feed velocities and the concentrations of the NaCl solution, the simulated permeate flowrate was compared with the experimentally obtained data.

## 3. Results and Discussion

### 3.1. Model Validation

A comparison between simulated permeate flowrate and experimentally measured data showed that the inconsistencies were less than 5% across three different experimental conditions (Table 1). This suggests that the CFD approach used in this investigation can capture the fluid flow and transport phenomena with a high degree of accuracy. 

### 3.2. Impact of Feed Spacer on Lab Scale Flat Sheet Module

**Figure 4:**
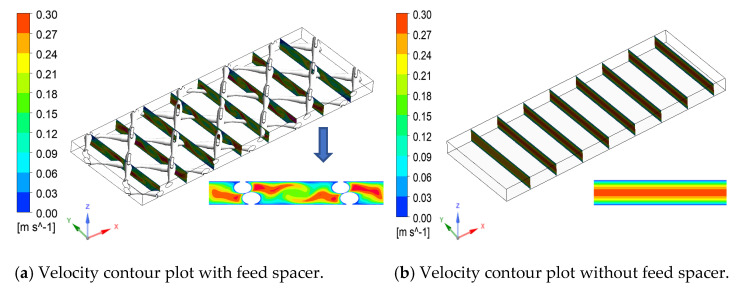
Velocity contour plot with the feed spacer (**a**) and without the feed spacer (**b**).

A wave-like flow pattern was observed in the flow field due to the presence of the spacer filaments (Figure 4a). Strong vortices appeared between the spacer filament and the membrane surface, which enhanced the flow velocities on the membrane surface (in the Y direction). These vortices disappeared in the absence of the spacer and led to turbulent flow in the middle of the channel (in bulk solution) while stagnant flow in the boundary layer where mass transfer relies on diffusion (Figure 4b). The presence of the counter-flow vortices inside the spacer-filled channel reduced the boundary layer thickness and consequently increased the average mass transfer coefficient to 1.59 × 10^−4^ compared to 7.95 × 10^−5^ in the absence of the spacer and reduced the CP. The mass transfer coefficient on the membrane surface can be calculated by the following:

(13) $$k=\frac{D}{\delta }$$

where k is the mass transfer coefficient, D is diffusion coefficient and δ is the thickness of the boundary layer.

**Figure 5:**
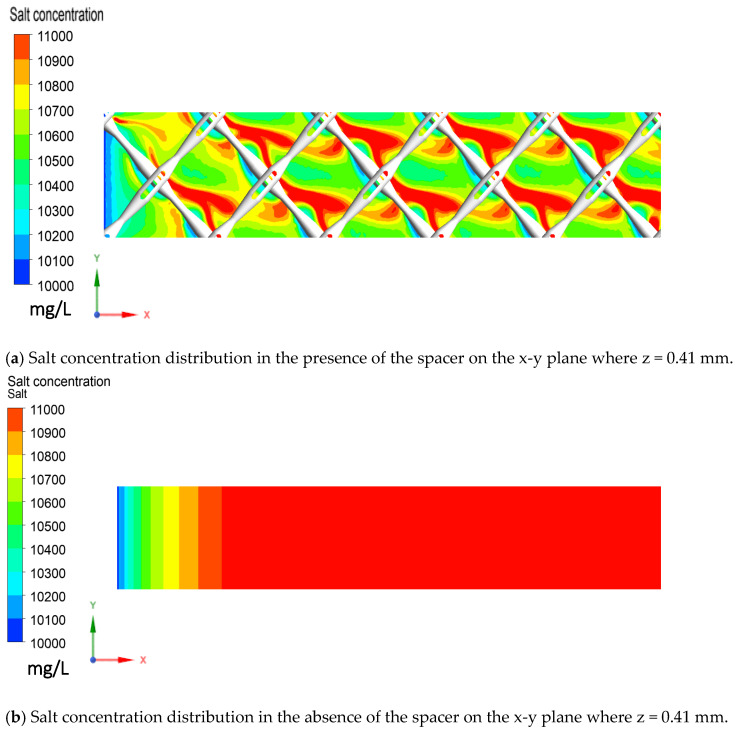
Salt concentration distribution on the x-y plane where z = 0.41 mm in the presence and absence of the feed spacer: (**a**) with the spacer; (**b**) without the spacer.

It is also interesting to observe that, in the presence of the spacer, the salt concentration in the feed channel was lower in the area that is further away from the spacer filaments while a higher concentration profile appeared over the back side of these filaments (Figure 5a). In contrast, salt concentration is uniform across the membrane surface in the absence of the spacer and increased steadily along the direction of the flow.

**Figure 6:**
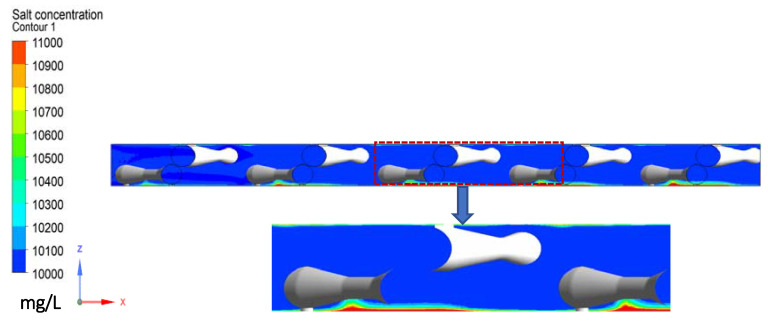
Salt concentration in the feed channel in the presence of the spacer at y = 1/2 of the width of the computational domain.

To further investigate the root cause of the CP phenomenon, an analysis was carried out on the cross section of the feed channel (the y-z plane). It can be seen that the feed spacer and the incoming flow created backflow vertically to the membrane surface, which enhanced the transfer of salt into the feed channel. Consequently, this greatly decreased the CP phenomenon at the flow facing side. In contrast, the local stagnant zone at the flow leaving side of the spacer increased the local concentration in the boundary layer and a stronger CP phenomenon was observed (Figure 6). This is consistent with the flow behaviour where higher vortices enhanced the back transport of salt away from the membrane surface. 

**Figure 7:**
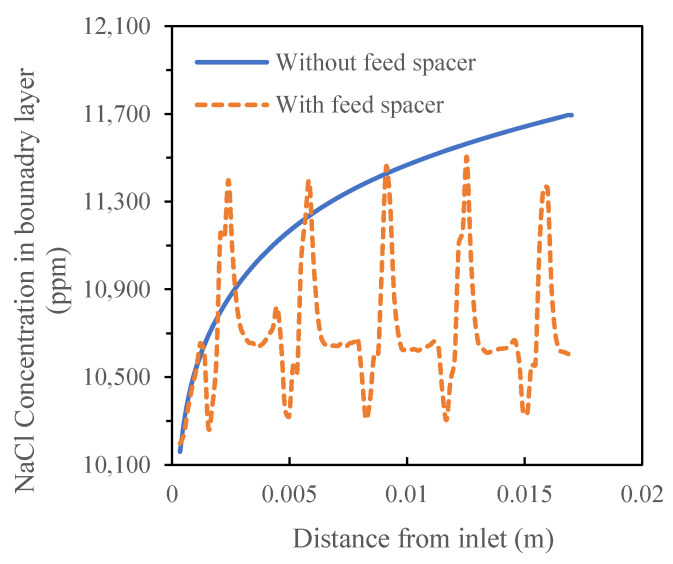
Salt concentration profiles in the boundary layer of the crossflow flat sheet module in the absence and presence of the feed spacer.

A quantitative analysis showed that the presence of the feed spacer led to a periodic fluctuation of salt concentration in the boundary layer between 10,196 mg/L and 11,504 mg/L while the salt concentration in the feed channel without a spacer increased along the flow direction from 10,195 mg/L to 11,694 mg/L (Figure 7). 

**Figure 8:**
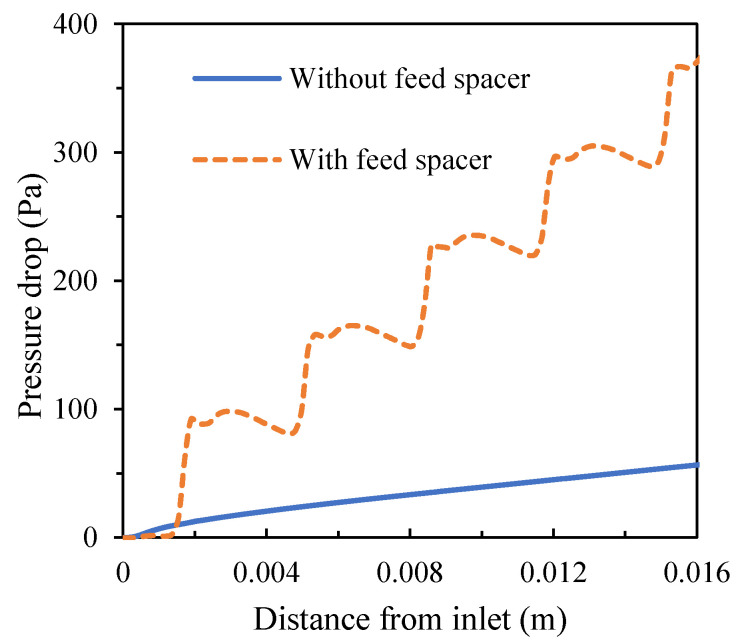
Pressure loss along the crossflow flat sheet module in the absence and presence of the feed spacer.

**Figure 9:**
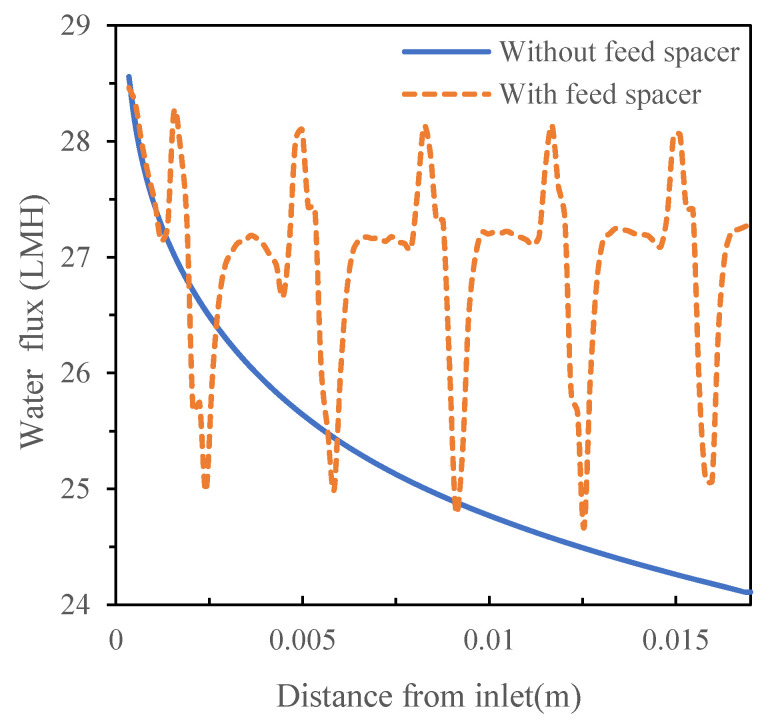
Permeate flux of the crossflow flat sheet module in the absence and presence of the feed spacer.

Although the spacer enhances mass transfer and creates higher turbulence, it also causes higher pressure drops, which is adverse to the filtration process. With the feed pressure of 1.55 MPa in the simulated conditions, the total pressure loss was approximately 369.75 Pa in the presence of the spacer compared to the total pressure loss of 56.5 Pa in the absence of the spacer (Figure 8). Taking into consideration the effects of mass transfer and pressure loss, total flux was 27.03 L/(m^2^ h) (LMH) in the presence of the spacer compared to 25.23 LMH in the absence of the spacer (Figure 9).

### 3.3. Impact of Spacer on Full-Size RO Module

**Figure 10:**
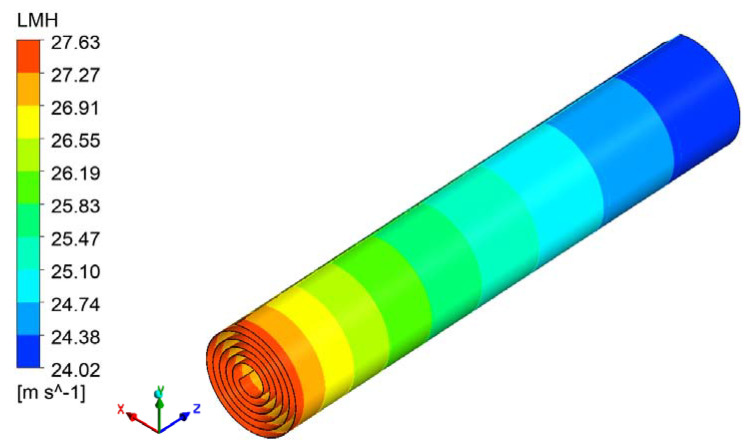
Permeate flux contour plot of the full-size spiral wound RO membrane module.

Simulations were conducted on the full-size RO module (Dupont Filmtec-BW30-400) with a feed pressure of 1.09 MPa. The simulation results on 10,000 mg/L of feed concentration showed that permeate flux reduced linearly along the feed direction in the absence of the feed spacer (from 27.6 LMH near the inlet to 24.1 LMH near the outlet) (Figure 10). The flow velocity had a minimal component that was perpendicular to the feed direction and tangential to the Archimedes curve; it had a magnitude of less than 0.003 m/s, which was only 1.5% of the average axial flow velocity. Thus, the flow velocity’s impact on the flow field was limited and this is consistent with previous researchers’ conclusion that the curvature of the membrane surface has a negligible effect on the flow distribution within the feed spacer via the simulation of a single feed spacer module [29,30].

**Figure 11:**
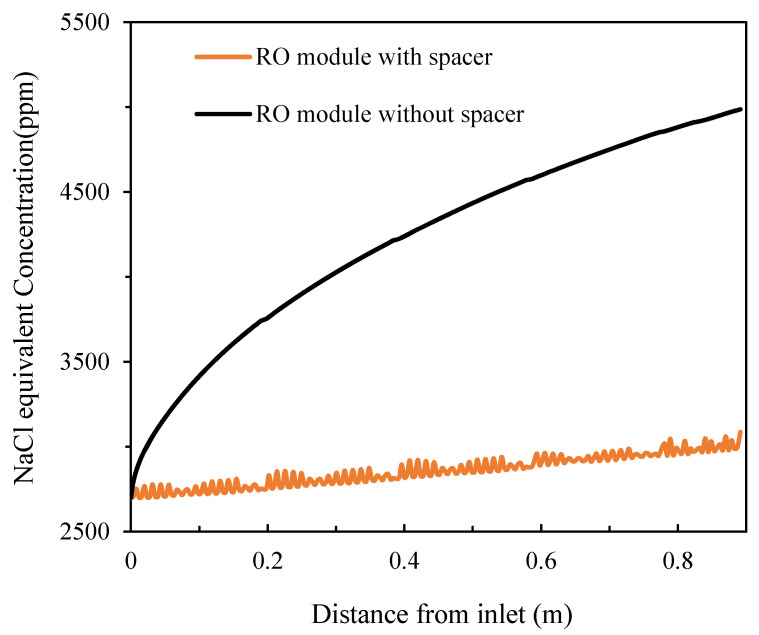
Salt concentration distribution of the spiral wound RO module along the feed direction in the absence and presence of the feed spacer.

**Figure 12:**
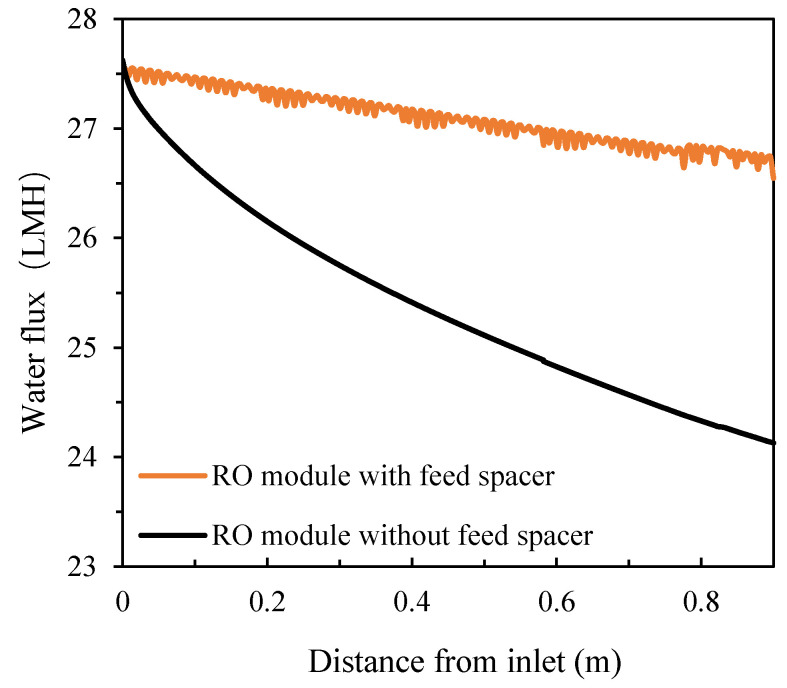
Flux distributions of the spiral wound RO module along the feed direction in the absence and presence of the feed spacer.

It can be observed that, for the full-size module, feed spacers played a more important role mitigating CP. Under the same feed concentration conditions (feed pressure of 1.09 MPa and salt concentration of 10,000 ppm), a severe concentration increase was observed (the outlet concentration increased by 84.67% compared to the inlet concentration) in the absence of the spacer while, in the presence of the spacer, the increase was only 15.30% (Figure 11). The corresponding fluxes decreased from 27.6 LMH at the inlet to 24.1 LMH at the outlet in the absence of the spacer while there was only a minor drop to 26.5 LMH in the presence of the spacer (Figure 12). 

## 4. Conclusions

A 3D CFD model was developed in this work to provide insights into the role of feed spacers in full-scale spiral wound RO modules. The mechanisms of how flow velocities and mass transfer differ in a fluid channel in the absence and presence of a feed spacer were presented. The variations in salt concentration and pressure drops caused by the spacer from the inlet to the outlet were quantified. This investigation provides the foundation for using 3D CFD tools for the design and optimization of full-scale RO modules. The accurate prediction of CP and flux profiles on the different locations of a membrane module could enable a better understanding of fouling potential and an improved module and spacer design. This investigation enables the evaluation and comparison of full-scale RO modules by different manufactures at different operating conditions, which is still a subject of on-going work.

## Figures and Tables

**Table 1:** Comparison of the simulation results with experimentally measured data at three different conditions.

Experiment Number	Feed Pressure (MPa)	Feed Flowrate (mL/min)	Feed Concentration, NaCl (mg/L)	Experimental Permeate Flowrate (mL/min)	Simulated Permeate Flowrate (mL/min)	Error
1	1.8	169.68	4509.4	4.67 ± 0.1	4.50	3.58%
2	1.4	177.24	4509.4	3.75 ± 0.08	3.67	2.08%
3	1.7	177.24	2089.6	6.44 ± 0.17	6.16	4.44%

## Data Availability

All data presented in this study are available in the current article.

## Nomenclature

A	permeability coefficient of water through the corresponding membrane (m·s^−1^·Pa^−1^)
a	effective membrane surface area for solution passage (m^2^)
B	permeability coefficient of inorganic salt (m·s^−1^)
*D*	diffusion coefficient (m^2^·s^−1^)
*δ*	boundary layer thickness (m)
$C_{m}$	concentration of the inorganic salt on the feed side of the membrane (kg·m^−3^)
$C_{p}$	concentration of the inorganic salt on the permeate side of the membrane (kg·m^−3^)
*K*	mass transfer coefficient
J	permeate flux, (m^3^·m^−2^·s^−1^)
$J_{s}$	salt flux (kg·m^−2^·s^−1^)
μ	fluid viscosity (kg·m^−1^·s^−1^)
ΔP	pressure difference across both sides of the membrane (Pa)
ΔΠ	osmotic pressure difference across both sides of the membrane (Pa)
p	pressure (Pa)
ρ	fluid density (kg·m^−3^)
*S*	source term
u	fluid velocity vector (m·s^−1^)
V	corresponding effective computational domain volume (m^3^)
v	flow velocity (m·s^−1^) (**u** is the vector)
